# Filiform polyposis with sigmoid colon adenocarcinoma: a case report

**DOI:** 10.1186/s40792-019-0747-x

**Published:** 2019-11-28

**Authors:** Takayuki Okuno, Takamitsu Kanazawa, Hirohisa Kishi, Hiroyuki Anzai, Koji Yasuda, Soichiro Ishihara

**Affiliations:** 1 Department of Surgery, Douai Memorial Hospital, 2-1-11 Yokoami, Sumida-ku, Tokyo, 130-8587 Japan; 2 Department of Pathology, Douai Memorial Hospital, 2-1-11 Yokoami, Sumida-ku, Tokyo, 130-8587 Japan; 3 Department of Surgical Oncology, The University of Tokyo, 7-3-1, Tokyo, Japan

**Keywords:** Filiform polyposis, Colon, Adenocarcinoma, Dysplasia, Inflammatory carcinogenesis

## Abstract

**Background:**

Filiform polyposis is a rare form of inflammatory polyposis, which is occasionally formed in the colon of patients with history of inflammatory bowel disease (IBD). It is characterized by presence of several to hundreds of slender, worm-like polyps in the colon lined by histologically normal colonic mucosa and often coalesce, resulting in a tumor-like mass. Filiform polyposis is most frequently associated with a post-inflammatory reparative process in patients with IBD history, and only cases of filiform polyposis occurring in patients without IBD history have been reported. Filiform polyposis has been considered as a benign inflammatory polyposis without any risk of dysplasia, while the possibility of carcinogenesis of inflammatory polyps is not fully excluded. To date, only three cases of filiform polyposis coexisting with dysplasia have been reported.

**Case presentation:**

A 59-year-old male patient with no past medical history of IBD underwent laparoscopic sigmoidectomy for obstructive filiform polyposis, which was associated with sigmoid colon adenocarcinoma. Based on the histological findings of the resected specimen, invasive sigmoid colon adenocarcinoma was surrounded by filiform polyposis, and adenocarcinoma also scattered uniformly on the surface of filiform polyposis. In immunohistochemistry, abnormal p53 expression was observed in adenocarcinoma, while it was not shown in mucosa on filiform polyposis.

**Conclusions:**

This is the fourth case of filiform polyposis that is closely associated with colon dysplasia or adenocarcinoma based on histological findings. However, immunohistochemical findings did not support the theory that inflammation initiates adenocarcinoma in filiform polyposis like IBD. Hence, further immunohistochemical and genetic analyses are needed to clarify the association between filiform polyposis and carcinogenesis.

## Background

Filiform polyposis is a rare form of inflammatory polyposis that is occasionally formed in the colon of patients with history of inflammatory bowel disease (IBD). Filiform polyposis is characterized by presence of several to hundreds of slender, worm-like polyps in the colon lined by histologically normal colonic mucosa [1]. Polyps often present as multiple mucosal projections that can reach up to 100 mm as in this case, and the term giant inflammatory polyposis has been used to describe these kinds of polyposis [2]. Filiform polyposis is most frequently associated with a post-inflammatory reparative process in patients with IBD history [3] and rarely associated with colon diverticulitis and intestinal obstruction in patients without IBD history [4, 5]. Filiform polyposis has been considered as a benign inflammatory polyposis without any risk of dysplasia [1], while the possibility of carcinogenesis of inflammatory polyps is not fully excluded [6]. To date, three cases with filiform polyposis coexisting with dysplasia have been reported [7–9]. Herein, we report a case of filiform polyposis that is closely associated with sigmoid colon adenocarcinoma in a 59-year-old patient without IBD history.

## Case presentation

A 59-year-old male patient was referred to our institution because of diarrhea and lower abdominal pain. He lost 6 kg of weight in 6 months. He did not have any personal or family history of polyps, colon cancer, and IBD. Physical examination revealed lower abdominal pain without tenderness and mass in the lower abdominal area. Blood examination revealed anemia (hemoglobin level 9.3 g/dl), hypoproteinemia (protein level 4.0 g/dl), and hypoalbuminemia (albumin level 1.2 g/dl). The carcinoembryonic antigen level was slightly increased (6.4 ng/ml).

**Fig. 1:**
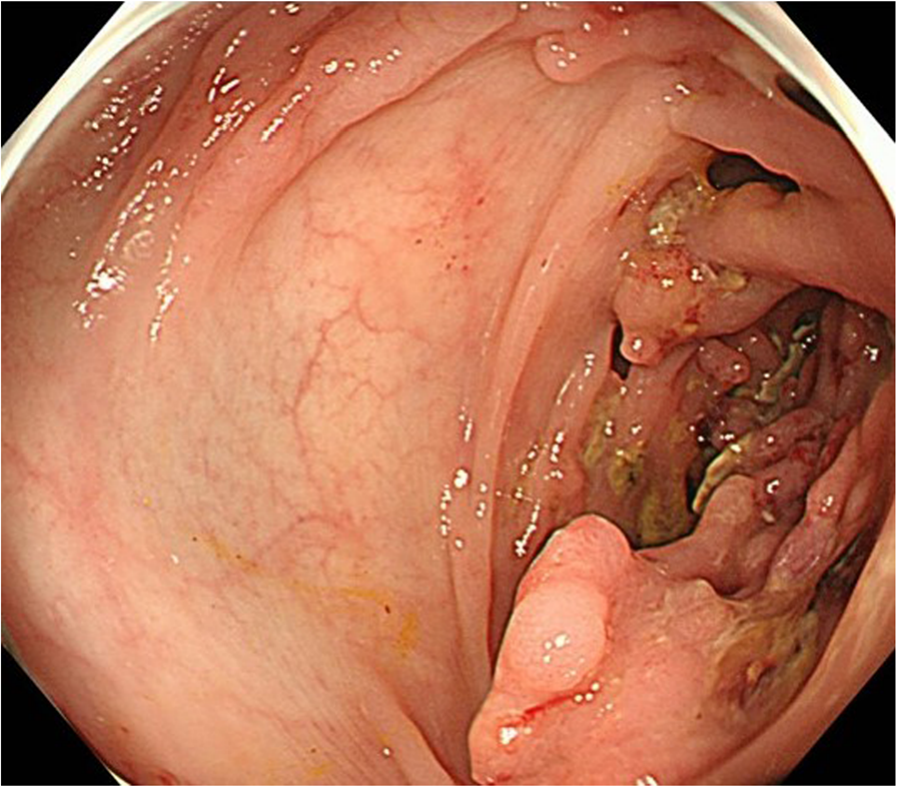
Colonoscopic study of the tumor. Colonoscopy revealed a large tumor with numerous white-pale reddish polyps of worm-like shape in the sigmoid colon. Biopsies showed well-differentiated to moderately differentiated tubular adenocarcinoma. The colonoscope could not be inserted beyond this point

Colonoscopy revealed a large tumor with numerous white-pale reddish polyps of worm-like shape in the sigmoid colon (Fig. 1). Biopsies of the tumor were performed, and histology of the examined sections indicated well-differentiated to moderately differentiated tubular adenocarcinoma. The colonoscope could not be inserted beyond this point; the colon distal to the tumor and rectum was normal. Computed tomography (CT) imaging showed an 80-mm heterogeneous tumor of sigmoid colon, which raised suspicion of invasion to the abdominal wall in the anterior side. Patient’s CT scan also showed a few amounts of abdominal dropsy, and no swelling of lymph nodes and no distant organ metastasis were shown. FDG-PET/CT denied the existence of other tumors in proximal portion of the colon beyond the tumor.

**Fig. 2:**
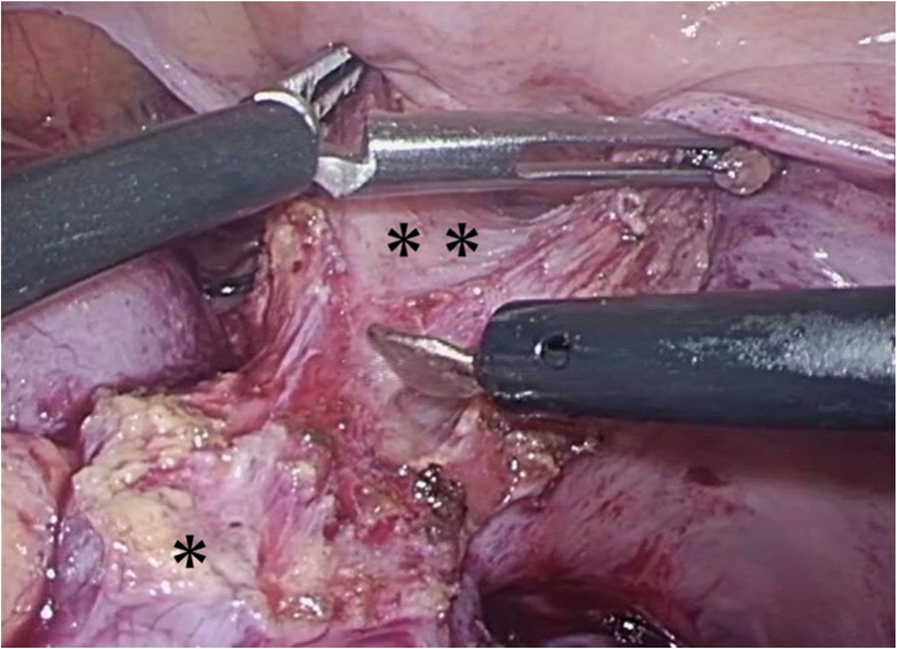
Laparoscopic study of sigmoid colon during operation. The tumor did not spread to the serosa of the sigmoid colon (asterisk), but the colon strongly adhered to the abdominal wall (double asterisk) based on the intraoperative findings. A part of the abdominal wall was also resected

Based on these findings, the patient underwent laparoscopic sigmoidectomy with level D3 lymph node dissection. The patient was placed in Trendelenburg position. Camera, three 5-mm and one 12-mm ports were placed in the umbilicus, bilateral upper, and lower abdomen. Intraoperative findings showed that the tumor did not spread to the serosa of the sigmoid colon, but the sigmoid colon strongly adhered to the abdominal wall. Therefore, a part of the abdominal wall was also resected (Fig. 2). The blood loss was 30 ml, and the total operative time was 226 min. His postoperative recovery was uneventful, and he was discharged 7 days after surgery. His condition remained stable and no recurrence was noted within 18 months.

**Fig. 3:**
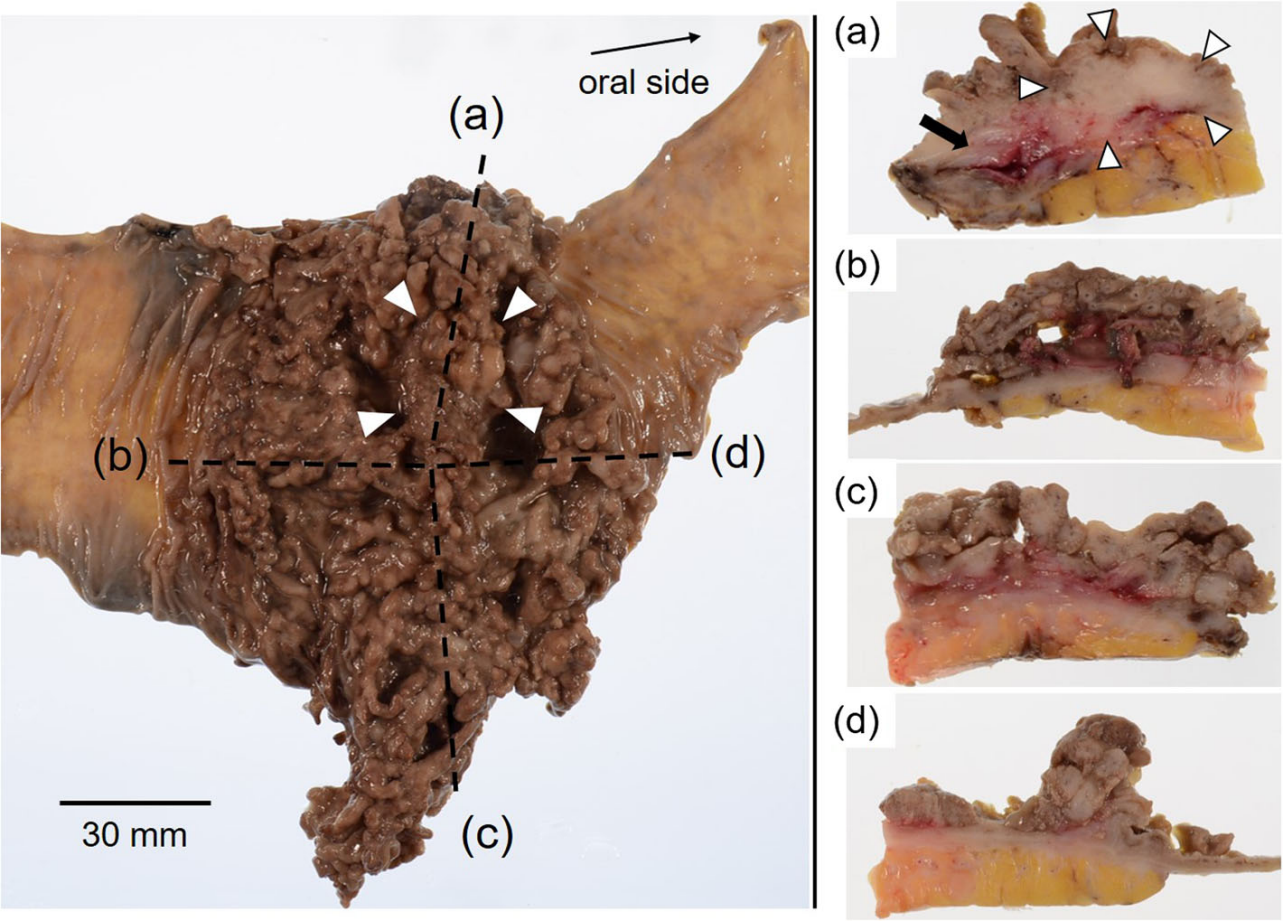
Macroscopic study of the surgical specimen after formalin fixation. Sigmoidectomy specimen showed a tumor measuring 110 × 95 mm; it had a coral-reef-like surface and with numerous soft, worm-like polyps. A solid 30-mm mass, surrounded by these polyps, developed on the anterior wall of the sigmoid colon (white arrowhead). **a** A solid mass did not spread to the serosa (white arrowhead). The anterior wall was thickened with several diverticulitis (black arrow). **b**–**d** The tumor was almost constituted with numerous soft, worm-like polyps. Histopathological findings of these polyps are shown in Fig. 4

The resected specimen was a 300-mm colonic segment that included a sigmoid colon and upper rectum. Macroscopically, a tumor measuring 110 × 95 mm was noted. The tumor had a coral-reef-like surface and was almost constituted with numerous soft, worm-like polyps. A solid 30-mm mass, which was surrounded by these polyps, was found on the anterior wall of the sigmoid colon. The anterior wall of the sigmoid colon was thickened with several diverticulitis, and an adhesion occurred at the resected part of the abdominal wall (Fig. 3).

**Fig. 4:**
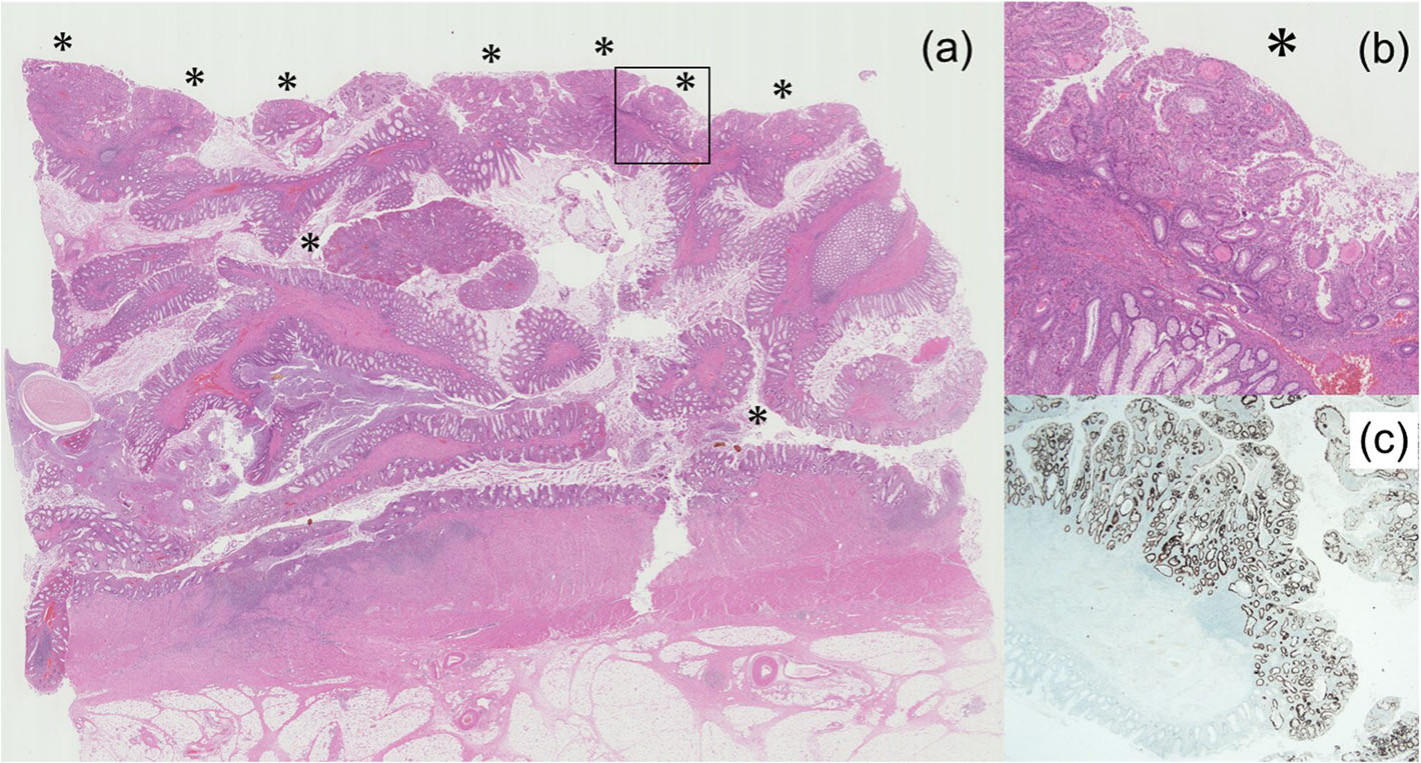
Histopathological study of filiform polyposis and adenocarcinoma (hematoxylin-eosin and p53-immunohistochemistry staining). Histopathological findings revealed that the worm-like polyps were filiform polyposis; dilated blood vessels, fibrovascular cores, and infiltrated neutrophils and lymphoid cells were seen in submucosal tissue of the polyp axis (**a**, × 5). Furthermore, well-differentiated to moderately differentiated tubular adenocarcinoma developed on the filiform polyposis. They mostly existed in the intramucosal area and were scattered uniformly on the surface of filiform polyposis (asterisks). The square part of Fig. 4a. The adenocarcinoma developed in the intramucosal area of the filiform polyposis (**b**, × 50). Immunohistochemistry using anti-p53 verified that the normal mucosa of filiform polyposis absence of abnormal p53 expression, while the adenocarcinoma on filiform polyposis showed an abnormal p53 nuclear expression (**c**, × 50)

Histopathological findings revealed that the worm-like polyp was an inflammatory polyp; dilated blood vessels, fibrovascular cores, and infiltrated neutrophils and lymphoid cells were found in the submucosal tissue of the polyp axis. These findings led to the diagnosis of filiform polyposis. Furthermore, well- to moderately differentiated tubular adenocarcinoma developed on the inflammatory polyps with erosive mucosa. These adenocarcinomas mostly existed in the intramucosal area and were scattered uniformly on the surface of filiform polyposis. The solid mass on the anterior wall also showed a well- to moderately differentiated tubular adenocarcinoma, which invaded the subserosa with vascular invasion. No lymph node metastasis was noted, and no adenomatous component was found in the tumor. Immunohistochemistry using anti-Ki-67 and anti-p53 verified that the erosive mucosa of filiform polyposis showed neither proliferation abnormality nor p53 abnormal expression. On the contrary, the adenocarcinoma on filiform polyposis and the mass on the anterior wall showed an abnormal p53 nuclear expression (Fig. 4). Finally, histopathological examination of the resected specimen led to the diagnosis of filiform polyposis and sigmoid colon adenocarcinoma pT3N0M0 StageIIA [10].

## Discussion

The term filiform polyposis was first coined by Appelman in 1984, who used it to describe a syndrome involving the radiographic appearance of numerous long slender worm-like defects in the colon with a normal haustral pattern [11]. Filiform polyposis is known as a rare inflammatory polyposis that occurs more frequently in patients with IBD history. Histologically, a polyp is characterized by dilated blood vessels and fibrovascular cores in submucosal tissues and covered with normal mucosa. Polyps vary greatly in number, from several to hundreds, and often coalesce, resulting in a tumor-like mass as in this case. Other terms, such as giant inflammatory polyposis and giant pseudopolyposis, have been used to describe these kinds of polyposis [2, 12]. The most common sites affected are the sigmoid colon and rectum [13], but polyposis can extend as far as the cecum and rarely occur in the stomach and small bowel [1].

In patients with IBD, long-term inflammation of the colonic mucosa with alternative periods of ulceration and healing is believed to be a prerequisite for the development of these worm-like polyps [3, 11]. Recently, rare cases of filiform polyposis in patients without history of IBD have been reported. In these cases, inflammatory cytokines [4] and the traction of redundant mucosa by intestinal peristalsis with hyperplasia and fibrosis of the intestinal wall, induced by colon diverticulitis, might relate to the development of filiform polyposis [5]. Furthermore, filiform polyposis developed due to a circumferential tumor obstruction in the colon [14]. In our case, hyperplasia of intestinal wall with sigmoid colon diverticulitis and tumor obstruction was observed in patients without IBD history. These findings are possibly associated with the development of filiform polyposis.

Filiform polyps are occasionally difficult to distinguish from “filiform serrated adenoma” based on their endoscopic appearance; hence, a biopsy or polypectomy is necessary to confirm the exact diagnosis [15]. Biopsy is also important for numerous conglomerated polyps as in this case. When biopsy proved adenoma or adenocarcinoma, polyps should be removed through a colonoscope or surgical colectomy [1]. When patients with filiform polyposis experiences symptoms such as bleeding and obstruction, the polyps should be removed [16]. Furthermore, the presence of occult dysplasia is suspected [7, 8]. In our case, biopsy for obstructive filiform polyposis showed adenocarcinoma; hence, laparoscopic sigmoidectomy with level D3 lymph node dissection was performed. The resected specimen showed invasive sigmoid colon adenocarcinoma surrounded by filiform polyposis.

Filiform polyposis has been considered as a benign inflammatory polyposis without any risk of dysplasia [1], while the possibility of carcinogenesis of inflammatory polyps is not fully excluded [6]. In our patient, invasive sigmoid colon adenocarcinoma coexisted with filiform polyposis, and adenocarcinoma also developed on the surface of filiform polyposis: histological findings showed the association between filiform polyposis and adenocarcinoma. To date, only three cases of tumor coexisting with filiform polyposis and dysplasia have been reported, which were similar to our case [7–9]. Boulagnon reported the case of filiform polyposis coexisting with mucinous ascending colon adenocarcinoma. Tubular adenoma and high-grade dysplasia also developed on filiform polyps. Molecular analysis of adenoma and adenocarcinoma revealed microsatellite stable status and absence of BRAF mutation; meanwhile, immunochemistry showed abnormal p53 expression in the adenoma and adenocarcinoma, but this was not observed in the mucosa of the polyps [9]. In our case, immunohistochemistry using anti-p53 and anti-Ki67 revealed that the erosive mucosa on filiform polyps showed neither proliferation abnormality nor p53 abnormal expression, while the adenocarcinoma showed an abnormal p53 nuclear expression. Since p53 mutations were observed in the inflamed mucosa in the early stage of inflammatory carcinogenesis [17], these findings do not suggest inflammatory carcinogenesis of filiform polyposis. Furthermore, the tumor found in our patient exhibited the same type of dysplasia: this finding was more suggestive of an incidental combination of two distinct pathological entities than carcinogenesis from inflammatory polyps.

Hence, we suggested the following model of tumor development in our case. First, sigmoid colon adenocarcinoma subcircumferentially existed; then filiform polyposis was formed by an inflammation of colon diverticulitis and the obstruction. Second, filiform polyposis was formed by an inflammation of colon diverticulitis; then the obstruction caused an implantation of sigmoid colon adenocarcinoma to erosive mucosa of filiform polyposis. Colon adenocarcinoma rarely develops in the defective part of mucosa [18]. Third, collision between sigmoid colon adenocarcinoma and the malignant transformation of filiform polyposis possibly occurred. Either way, the coexistence of advanced cancer and widely spreading mucosal cancer could not be fully explained.

## Conclusion

This is the fourth case of filiform polyposis that is closely associated with colon dysplasia or adenocarcinoma. However, immunohistochemical analysis of p53 mutation did not support the theory that inflammation triggered the occurrence of adenocarcinoma in filiform polyposis. Hence, immunohistochemical and genetic analyses are needed to clarify the relationship between filiform polyposis and carcinogenesis.

## Data Availability

All data generated or analyzed during this study are included in this published article.

## Abbreviations

CT: Computed tomography
IBD: Inflammatory bowel disease
